# Evaluation of an exposure assessment used in epidemiological studies of diesel exhaust and lung cancer in underground mines

**DOI:** 10.3109/10408444.2012.689755

**Published:** 2012-03-09

**Authors:** Kenny Crump, Cynthia Van Landingham

**Affiliations:** 1 Louisiana Tech University, Ruston, LA, USA; 2 ENVIRON International Corporation, Monroe, LA, USA

**Keywords:** Exposure assessment, underground mines, diesel exhaust, carbon monoxide, respirable elemental carbon

## Abstract

NIOSH/NCI (National Institute of Occupational Safety and Health and National Cancer Institute) developed exposure estimates for respirable elemental carbon (REC) as a surrogate for exposure to diesel exhaust (DE) for different jobs in eight underground mines by year beginning in the 1940s—1960s when diesel equipment was first introduced into these mines. These estimates played a key role in subsequent epidemiological analyses of the potential relationship between exposure to DE and lung cancer conducted in these mines. We report here on a reanalysis of some of the data from this exposure assessment. Because samples of REC were limited primarily to 1998–2001, NIOSH/NCI used carbon monoxide (CO) as a surrogate for REC. In addition, because CO samples were limited, particularly in the earlier years, they used the ratio of diesel horsepower (HP) to the mine air exhaust rate as a surrogate for CO. There are considerable uncertainties connected with each of these surrogate-based steps. The estimates of HP appear to involve considerable uncertainty, although we had no data upon which to evaluate the magnitude of this uncertainty. A sizable percentage (45%) of the CO samples used in the HP to CO model was below the detection limit which required NIOSH/NCI to assign CO values to these samples. In their preferred REC estimates, NIOSH/NCI assumed a linear relation between C0 and REC, although they provided no credible support for that assumption. Their assumption of a stable relationship between HP and CO also is questionable, and our reanalysis found a statistically significant relationship in only one-half of the mines. We re-estimated yearly REC exposures mainly using NIOSH/NCI methods but with some important differences: (i) rather than simply assuming a linear relationship, we used data from the mines to estimate the CO—REC relationship; (ii) we used a different method for assigning values to nondetect CO measurements; and (iii) we took account of statistical uncertainty to estimate bounds for REC exposures. This exercise yielded significantly different exposure estimates than estimated by NIOSH/NCI. However, this analysis did not incorporate the full range of uncertainty in REC exposures because of additional uncertainties in the assumptions underlying the modeling and in the underlying data (e.g. HP and mine exhaust rates). Estimating historical exposures in a cohort is generally a very difficult undertaking. However, this should not prevent one from recognizing the uncertainty in the resulting estimates in any use made of them.

## Introduction

Earlier epidemiological studies of the possible relationship between diesel exhaust (DE) and lung cancer focused on occupations in which the workforce worked in proximity to operating diesel engines, such as railroad workers and truck drivers (Garshick et al., 1988, 2004, 2008; Steenland et al., 1992). Criticisms of these and other studies include potential confounding and particularly the lack of reliable historical data on exposure to DE (Attfield et al., 2012). In response, the National Cancer Institute and the National Institute of Occupational Safety and Health (NCI/NIOSH, 1997) proposed that a study be conducted in noncoal, nonmetal underground mines that used diesel equipment, where air contaminants such as coal dust, methane, radon and metal contamination would not be present, so that emissions from diesel engines would be essentially the only air pollutants present. The ambient levels of diesel PM in some mines were known to be quite high – perhaps an order of magnitude higher than levels in previously studied cohorts composed of railroad workers and truck drivers.

Accordingly, NIOSH and NCI (referred to hereafter as NIOSH/NCI) selected eight mines (Mine A, limestone; Mines B, D, and J, potash; Mine E, salt; and Mines G, H, and I, trona) and conducted a historical cohort study (Attfield et al., 2012) and a nested case control study (Silverman et al., 2012) of workers in these mines regarding the potential relationship between their DE exposure and lung cancer. Historical estimates of DE exposures of these workers were obtained from an assessment of DE exposures conducted in the eight mines (Stewart et al., 2010; Coble et al., 2010; Vermeulen et al., 2010a, 2010b). This assessment provided job-specific yearly historical estimates of respirable elemental carbon (REC), selected as a surrogate for DE, beginning with the year in which diesel equipment was first introduced into a mine (1947–1967), through 1998–2001.

The subject of this article is the NIOSH/NCI exposure assessment, which played a critical role in the subsequent epidemiological studies. Data used in the NIOSH/NCI exposure assessment were provided to the Mining Awareness Resource Group (MARG) who subsequently provided them to us and asked us to critique the assessment. Details regarding the eight mines, the data upon which the NIOSH/NCI exposure assessment was based, and the methods employed, may be found in the above referenced papers.

## Overview of NIOSH/NCI data and methods

Table 1 is a reproduction of Table 2 of Stewart et al. (2010) that summarizes the diesel-related exposure data collected by NIOSH/NCI from the eight mines that are the subject of their study. The shaded part of the table shows the data that were ultimately relied upon by NIOSH/NCI and provided to MARG. As Table 1 shows, samples of REC were mainly available only from a survey (DEMS) conducted during 1998–2001 as part of the NIOSH/NCI study. To estimate historical exposures to REC, NIOSH used samples of carbon monoxide (CO) as a surrogate for REC, using the 1998–2001 data from DEMS to establish a relationship between CO and REC. However this calculated CO to REC relationship was not used in establishing the preferred estimates of REC exposures; instead a linear relationship between CO and REC was assumed. CO samples were very limited prior to 1976 (Table 1), whereas the eight mines began using diesel equipment much earlier—between 1947 and 1967. To fill this gap in the CO data, NIOSH/NCI used historical information on diesel equipment used at the mines to estimate the total horsepower (HP) by year of diesel equipment in each mine, adjusted for the amount of time each piece of equipment was estimated to have been used. This information was used, along with similar information on the rate of mine air exhaust (CFM) to establish a relationship between adjusted HP, CFM and other determinants, and CO levels through a regression analysis. This relationship was used to estimate CO levels in each mine by year. These estimated CO levels were converted into yearly estimates of job-specific exposures to REC using the job-specific REC exposures estimated using the 1998–2001 data and the assumed linear relationship between CO and REC.

The construction of the HP database used in this analysis was described by Vermeulen et al. (2010b) as follows:

HP of the diesel-powered equipment was available on inventories of diesel-powered equipment used underground, extending back as far as the early 1970s from the facilities. Inventories generally were available for a few years in the 1970s and the 1990s but rarely in the 1980s. The lack of inventories was compensated by a careful scrutiny of each mine’s production characteristics, trends over time in the number of diesel pieces used (for all the facilities, there was generally little change in equipment from year to year), and the number of years equipment was used, as well as being supplemented by information from the interviews. The specific section of the mine where the equipment was used was usually not identified. HP was directly available for 80% of the diesel equipment. For the other 20%, HP was estimated based on the same or similar equipment purchased about the same time in the same or in other facilities. From this information, the annual sum of the HP of all diesel-powered equipment in each mine was calculated based on all diesel engines used in a particular year. Vermeulen et al. (2010b)

A similar approach was used to estimate air exhaust rates by year:

Annual facility-specific estimates of total airflow rates exhausted from all operations within each underground mine, in cubic feet per minute (CFM) (1 CFM = 0.0283 m^3^ min^−1^), also were compiled. Records were available identifying the average total airflow rates exhausted from the underground operations when each exhaust shaft was installed since the beginning of diesel use and occa sionally for other years. Information was not, however, available for sections of the underground mine. Vermeulen et al. (2010b)

## Data available for this critique

Among the diesel-related exposure data listed in Table 1, we were provided with only the data from the DEMS survey and data on CO from other surveys (shaded in Table 1). In particular, we were not provided with data on CO_2_, NO, or NO_2_ other than in the years 1998–2001, which prevented us from studying the suitability of any of these emission constituents as alternative surrogates for diesel to replace CO. Some of the shaded data in Table 1 had been culled as we were provided with only 9366 CO measurements, compared to 11,170 listed in Table 1. This discrepancy could be due to the removal of samples collected using passive samplers, which Vermeulen et al. (2010a) state were not used in their analysis due to an increased number of such measurements being below the detection limit. Of the total of 9366 CO measurements, all were made underground except for 35 surface samples collected as part of the DEMS survey during 1998–2001. A large fraction of these CO measurements were below the detection limit (23 of 35 (66%) of above ground measurements and 4200 (45%) of 9331 underground measurements), and values were imputed (assigned) by NIOSH/NCI to these samples using a statistical methodology. For these we have only the imputed values and an indication that they were nondetects; we were not provided with the sample-specific detection limits (although median detection limits for specific surveys are listed in the publications). The imputed values provided to us were not the ones used in the published analysis, but had been reimputed. In addition to the sample values, we were provided with the year each sample was collected, the mine in which it was collected, and a code for location within that mine. For the personal samples collected in the DEMS survey, we were provided with job codes that could be linked to information indicating the percentage of time persons working in these jobs were estimated to have spent in various locations of the mine and above ground. We did not receive descriptions of the type of work represented by these job codes but we could determine a few of them by comparing our results with those in Coble et al. (2010).

The information we received regarding the HP and mine exhaust rates consisted of mine- and year-specific estimates of HP, adjusted for usage, HP from diesel equipment purchased after 1990 (to account for potentially less CO emission from this equipment), and air exhaust rates. No information was provided that would give objective evidence on the uncertainty in these estimates.

**Table 1:** Table 2 of Stewart et al. (2010) showing the number of area and personal DE-related measurements available to NIOSH/NCI by agent for the eight mining facilities.

Table 2. Number of area and personal DE-related measurements by agent for the eight mining facilities													

	Survey^a^												
		
	MIDAS 1976–2001		DEMS 1998–2001		MESA/BoM 1976–1977		Feasibility study 1994		Other 1954–1996		All surveys		
							
Agent	Are^b^	Personal^b^	Area	Personal	Area	Personal	Area	Personal	Area	Personal	Area	Personal	Total
CO	9746	46	208	0	1099	0	25	0	46	0	11,124	46	11,170
CO_2_	8234	15	390	0	961	0	17	0	49	0	9651	15	9666
NO	45	0	381	995	24	0	42	69	9	0	501	1064	1565
NO_2_	4288	38	387	1031	252	646	42	69	76	11	5045	1795	6840
TD	1	782	215	0	161	667	32	0	69	703	478	2152	2630
RD	0	324	209	2	99	0	31	0	158	178	497	504	1001
SD	0	0	121	0	0	0	69	0	20	0	210	0	210
TEC	0	0	224	0	0	0	0	0	0	0	224	0	224
REC	0	0	216	1156	0	0	0	69	12	4	228	1229	1457
SEC	0	0	209	0	0	0	0	0	0	0	209	0	209
TOC	0	0	224	0	0	0	0	0	0	0	224	0	224
ROC	0	0	221	1151	0	0	0	0	0	0	221	1151	1372
SOC	0	0	207	0	0	0	0	0	0	0	207	0	207
DPM/SCD	0	0	212	0	0	0	0	0	180	102	392	102	494
Total	22,314	1205	3424	4335	2596	1313	258	207	619	998	29,211	8058	37,269

The shaded areas indicate data, some of which was made available for our analysis. DPM, diesel particulate matter; RD, respirable dust; ROC, rcspirable organic carbon; SCD, submicron combustible dust; SD, submicron dust; SEC, Submicron elemental carbon; SOC, submicron organic carbon; TD, total dust, TEC, total elemental carbon; TOC, total organic carbon.

^a^ Surveys: the MSHA MIDAS (1976–2001); the DEMS (1998–2001) (Coble et al., 2010; Vermeulen et al., 2010b); the MESA/BoM (1976–1977) (Sutton et al., 1979); the feasibility study for the DEMS in Facility B (1994) (Stanevich et al., 1997): compliance visits by the State of New Mexico, MSHA hard copy reports, and the mining facilities (1954–1996).

^b^ Area measurements: personal measurements. The number includes both full-shift and short-term measurements.

**Table 2:** Comparison of our results of modeling the relationship between CO and REC (Equation (1)) with those of NIOSH/NCI.

Model	AIC^a^	β (slope)	Source
Fixed common intercept, fixed common slope	586.6 586.5 579.9	0.47 0.47 0.43	Our results NIOSH-NCI^b^ using our imputed values
Fixed mine-specific intercepts, fixed common slope	519.4 510.0	0.44 0.40	Our results using our imputed values
Fixed mine-specific intercepts, random mine specific slopes	522.7 516.8^c^ 512.6	Our results NIOSH-NCI using our imputed values
Fixed mine-specific intercepts, random common slope	524.2 516.8^c^ 514.9	0.43 0.58^d^ 0.39	Our results NIOSH-NCI using our imputed values

^a^ Measure of model fit (smaller indicates better fit). Our estimates of AIC and slope come from the maximum likelihood fitting alternative in the MIXED Procedure (SAS).

^b^ These results are from a model that is only described by NIOSH/NCI as the “regression model” but we assume from the similarity of results that they are referring to this model.

^c^ It is not clear to us whether this AIC was calculated using a common random slope or mine-specific random slopes.

^d^ It is not clear to us whether this slope was calculated using this model (preferred approach) or represents an average of mine-specific random slopes (not preferred).

## Our work

Our work initially focused upon reproducing some of the analyses reported in the four papers on the exposure assessment (Stewart et al., 2010; Coble et al., 2010; Vermeulen et al., 2010a, 2010b) to make sure we were interpreting the data and their analyses correctly. For example we were able to reproduce reasonably well results contained in Figure 1 of Stewart et al. (2010), Tables 1, 4 and the table in the Appendix of Coble et al. (2010), Table 1 and Figure 2 of Vermeulen et al. (2010a), and Tables 1 and 2 and Figure 2 from Vermeulen et al. (2010b). We would not expect to get exact matches in many cases due to differences in imputed data.

### Assigning 1998–2001 REC exposures to job codes

NIOSH/NCI used the data gathered from the 1998 – 2001 DEMS survey (Table 1) to assign REC exposures during those years to job codes (Coble et al., 2010). This was accomplished by assigning four groups of mine-specific personal underground REC measurements to each job code: Group U1 ⊆GroupU 2 ⊆GroupU 3 ⊆GroupU 4. Group U1 consisted of the measurements collected on workers with that specific job code; Group U2 consisted of measurements from job titles grouped based on the usual percentage of the work shift (<30%, 30–59%, and >59%) spent in each of four underground areas (i.e. face, haulage and travel ways, shop and offices, and, in three mines, crusher); Group U3 combined various Group U2 groups based on similarities in historical CO levels measured in these underground areas; and Group U4 consisted of all underground personal REC measurements from a mine. A job code was assigned the arithmetic mean (AM) of the REC measurements in the smallest numbered group that had at least five measurements. In addition, NIOSH/ NCI defined overrides which are exposures assigned to job codes in which NIOSH/NCI believed the AM was not consistent with the job description (Group U5). Although it is not clear to us how override exposures were assigned^1^, NIOSH/NCI did provide us with the override job codes and the exposures assigned to them. Thus, except for the job codes assigned an override exposure, the REC level for 1998–2001 assigned to each job code was based on at least five measurements. Table 2 of Stewart et al. (2010) indicates that 40% of exposure years of underground work in the mines were assigned REC exposures from Group U1, 40% from Group U2, 6% from Group U3, 12% from Group U4 and only 1% were assigned override exposures (Group U5).

**Figure 1:**
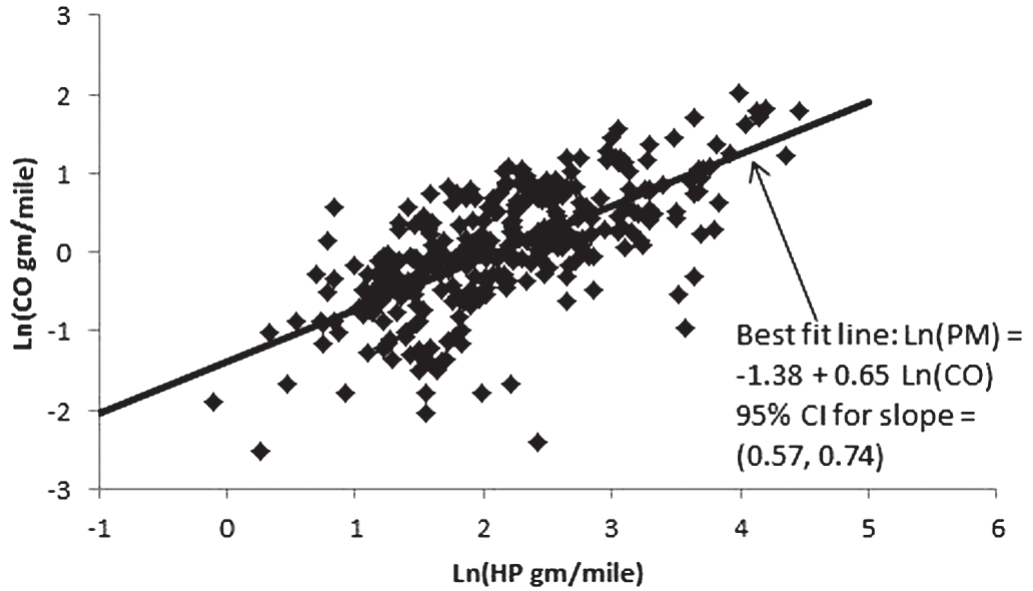
Linear fit to Ln(PM) versus Ln(CO) data in Yanowitz et al. (2000) shows a best slope of 0.65 and the confidence interval does not include the slope of 1.0 assumed by NIOSH/NCI.

**Figure 2:**
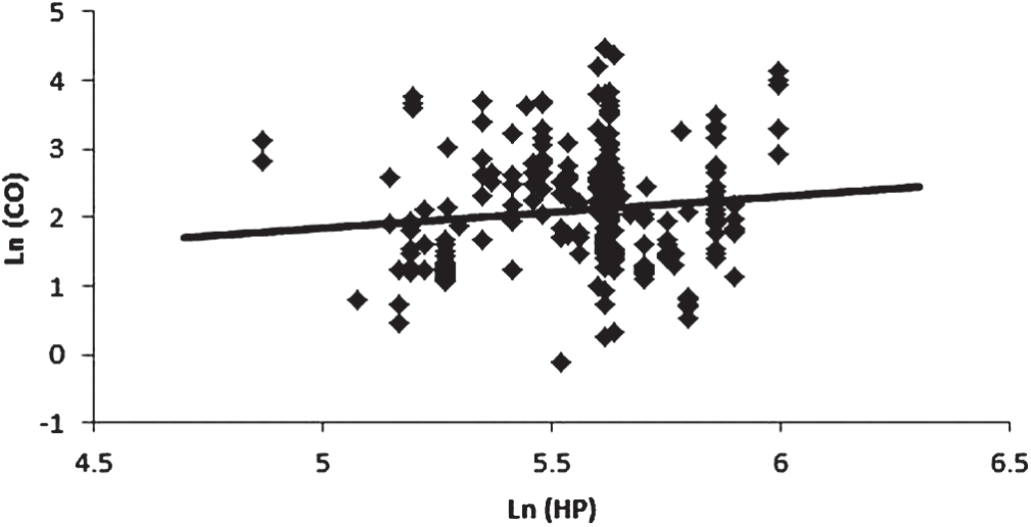
Graph of data from Yanowitz et al. (2000, Table 2) of Ln (CO) versus Ln (engine HP) with regression line showing a barely statistically significant relationship (*p* = .05, *r*^2^ = 0.01).

Surface jobs were divided into three groups based on their expected relative exposure intensity determined from expected proximity of the job in relation to the location of diesel-powered equipment. If a job group at a mine had at least five personal REC samples collected during the DEMS survey, the AM of these samples was used to represent REC exposures for jobs in that group at that mine. If there were fewer than five samples, the AM of personal samples of this group from all mines of the same type (e.g. potash, salt, trona, or limestone) was used, provided this expanded sample had at least five samples. Otherwise, the AM of samples from this job group from all mines was used. Surface and underground REC estimates were combined to estimate REC exposures in jobs that involved both surface and underground work.

We were able to reproduce the information in the published papers that was provided on these assignments in most cases (Table 4 and the Appendix of Coble et al., 2010). Although we did not receive job descriptions to go with the job codes, we were able to predict a few of them (25 out of a total of 492) by matching our results with those of Coble et al. (2010).

There were no REC samples for Mine J as this mine closed in 1993, before the DEMS survey was conducted. Consequently, personal job-specific REC exposures for 1993 for Mine J were estimated using data from Mine B. Mine B was selected “because of the similarity of geographic location, the type of mining [both were potash mines], the amount of HP present, and the air flow rates in the two facilities” Stewart et al. (2010).

### The relationship between REC and CO in the DEMS data (1998–2001)

NIOSH/NCI used the 1998–2001 DEMS underground area sampling data to establish a statistical relationship between REC and CO of the form


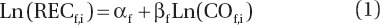


where f indexes mine and i indexes the measurements within a mine. They considered several versions of this model, including one in which αand β are both modeled as fixed effects, and one in which α is modeled as a fixed effect and β is modeled as a random effect. NIOSH/NCI preferred an overall slope (not mine specific) of β = 0.58 based on fixed mine-specific intercepts αand random β, because of the smaller AIC (a measure of goodness of fit in which smaller values indicate a better fit) that was obtained (516.8 vs. 586.5 for a fixed mine-independent slope, β, and a fixed mine-independent intercept, α), and they used β = 0.58 to construct an alternative set of REC exposure estimates.

Table 2 shows the results reported by NIOSH/NCI, along with our attempt at reproducing their analyses. In addition to making runs using the imputed CO values provided to us, we also made runs using one set of CO values we imputed^2^ for nondetects. There are several points to make with respect to this table. First of all, we did not obtain a slope as large as the β = 0.58 reported by Vermeulen (2010a) in any of our analyses. Rather our slopes ranged from 0.39 to 0.43 in analyses intended to reproduce their slope of 0.58. The only possible basis for their slope of 0.58 that we could determine was that the average of the mine-specific random slopes in our analysis that also assumed fixed mine-specific intercepts was 0.56 (with the remaining discrepancy possibly due to use of different imputations). However, the average of the mine-specific values is not as reliable as the value obtained modeling a single slope for all mines (0.39—0.43) (Table 2), because the single slope adjusts for differences in the data from the different mines, such as the number of samples, whereas the average of mine-specific slopes does not.

Second, although NIOSH/NCI preferred a random model for slope based on AIC values, apparently they were comparing a model with random slopes using fixed mine-specific intercepts (AIC = 516.8) to a model assuming a single fixed intercept and a single fixed slope applied to all mines (AIC = 586.5). However, Table 2 shows that the reduction in AIC is mainly due to allowing the intercepts to be mine-specific and not to the use of random slopes. In fact the smallest AIC (510.0) came from the analysis that applied our imputed values to a model that assumed fixed mine-specific intercepts and a fixed common slope.

Third, we note that different imputed values can make a sizable difference in the estimated slope and the AIC. Our results for imputed values in Table 2 are for a single imputation and do not represent the range of values that could be obtained from different imputations.

It appears that in their calculation NIOSH/NCI also used a single imputation to calculate the slope, β. Given the indication from our analysis (Table 2) that the different imputations can give important differences in the estimated slope, we conducted a multiple imputation approach based on maximum-likelihood estimation to assign values for CO that were below the limit of detection. Following NIOSH/NCI, we used the paired area samples of REC and CO from the 1998–2001 DEMS study after eliminating the above ground samples, which left 167 paired REC and CO samples, of which 26 of the CO samples were nondetects. Using maximum likelihood we fit three distributions to the censored CO data: the Log-normal, the Gamma (Johnson et al., 1994) and the Weibull (Johnson et al., 1994). The Gamma distribution was determined to have the best fit (i.e. largest likelihood). Therefore the Gamma distribution was used to impute CO values to nondetects. Since we did not have access to detection limits for individual samples, the median detection limit of 0.3 ppm (Vermeulen, 2010b) was used for all nondetects. Similar to the approach in Lubin et al. (2004), we used maximum likelihood to fit the Gamma distribution to 100 bootstrap samples of censored CO data, thereby generating 100 sets of imputed CO data using the Gamma distribution. We then ran linear regression analyses on the 100 sets of imputed data, regressing Ln(CO) on Ln(REC) using fixed mine-specific intercepts and a common fixed slope, which was the model that gave the best fit in the earlier analysis (Table 2). These results were combined to derive the parameter estimates, standard errors, and 95% confidence intervals using PROC MIANALYZE (SAS). The resulting slope from this analysis was β = 0.30, 95% CI: (0.061, 0.54), compared to the value of 0.58 estimated by NIOSH/NCI.

Despite estimating β = 0.58 from their data, NIOSH/ NCI opted to use β = 1 (i.e. a linear relationship) in their preferred method for estimating historical REC exposures. This decision was supported entirely by reference to Yanowitz et al. (2000), as Stewart et al. (2010), Vermeulen et al. (2010a) and Vermeulen et al. (2010b) cite this article numerous times as their only justification for assuming a linear relationship (i.e. β = 1) between CO and REC. For example, Vermeulen (2010b) states: “Although there was good external evidence that a relative change in historical CO concentrations can be directly translated to an identical change in REC (Yanowitz et al., 2000),…”

**Figure 3:**
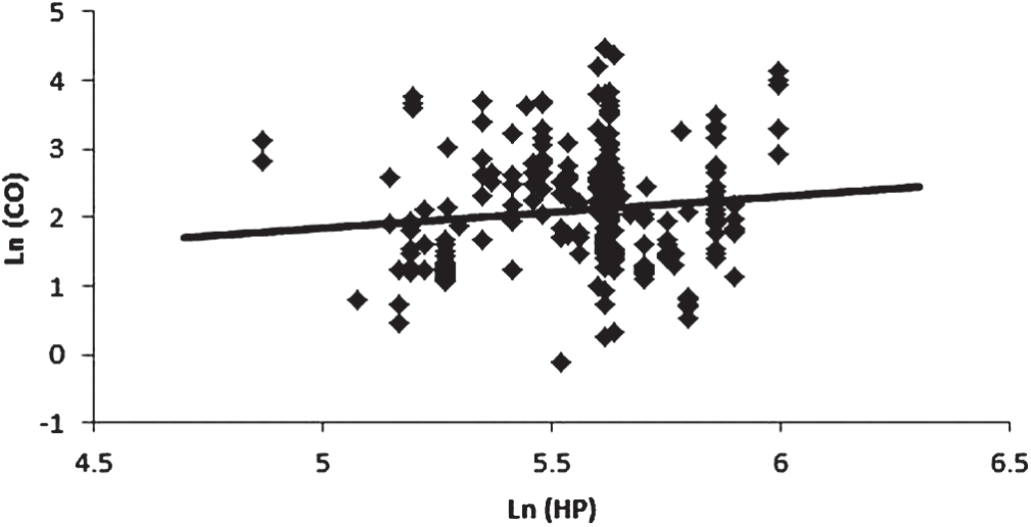
Graph of data from Yanowitz et al. (2000, Table 2) of Ln(PM) versus Ln(engine HP) with regression line showing no statistically significant relationship (*r*^2^ = 0.00001).

In reviewing Yanowitz et al. (2000), it was not clear to us what this conclusion (that REC varies linearly with CO) was based on unless it is Figure 3 from this article showing that both CO and particulate matter (PM) emissions in g/gal decreased at the same rate per year. We were unable to reproduce those rates, however, based on the data in Yanowitz et al., Table 2. Nevertheless, a more definitive evaluation of the relation between CO and PM can be obtained from a direct comparison of these emissions on a joint measurement basis. Accordingly, we conducted a linear regression of Ln(CO) on Ln(PM) (both PM and CO in units of g/mile) for the joint measurements from the diesel engines listed in Table 2 of Yanowitz et al. (2000) for which the required data are recorded (Figure 1). As indicated in Figure 1, the best fitting line has a slope of 0.65, 95% CI: (0.57, 0.74), and the confidence interval does not include the value of 1.0 assumed by NIOSH/NCI. Consequently, the claimed support from Yanowitz et al. for the assumed linear relationship between CO and REC appears to be questionable.

NIOSH/NCI also assumed a statistical relationship between Ln(HP) and LN(CO) (see next section), and the data in Table 2 of Yanowitz et al. (2000) can be used to test whether this assumption is reasonable. Figure 2 shows the results of a regression fit to the data points listed in Yanowitz et al. for which both HP and CO (g/mile) values are provided. The slope of the regression line is only borderline significant (*p* = .05) and the data indicate a large variation around the fitted line (*r*^2^ = 0.01, Figure 2).

Finally, the NIOSH/NCI estimation approach of estimating CO levels from HP and REC from CO implies that there is a relationship between HP and REC. This can also be tested directly using the data from Table 2 of Yanowitz et al. (2000). Figure 3 is a graph of Ln(PM g/mile) versus Ln(HP) for the measurements in Yanowitz et al., Table 2. A linear regression shows no relationship (*p* = .96, *r*^2^ = 0.0004, Figure 3).

In all of the analyses reported above for the data in Yanowitz et al. (2000), the results from vehicles tested more than once using the same test cycle, and without any additional mileage accumulated between tests, are averaged to produce a single data point (US EPA, 2002). We also repeated these analyses in which we averaged results for a test vehicle performed without any additional mileage over all test cycles and obtained even less evidence of relationships. For example, the relationship between Ln(CO) and Ln(PM) became nonsignificant (*p* = .19, *r*^2^ = 0.008) and the slope of the relationship between Ln(HP) and Ln(PM) became (nonsignificantly) negative.

### The historical relationship between CO and HP

Since there were very few CO measurements prior to 1976 (Table 1), to compensate for the lack of data at these earlier times and also at other times, NIOSH/NCI assumed a relationship between HP of diesel equipment used in the mines and CO levels. Relying on inventories of diesel-powered equipment used underground, extending back as far as the early 1970s, along with similar historical data on mine ventilation rates, they developed year- and mine-specific estimates of Adj HP (HP of underground equipment adjusted for assumed usage) and mine ventilation rates (CFM), beginning the first year diesel equipment was introduced into a mine and running through 1998–2001. To account for possible lower CO emissions from more modern diesel engines, they also developed a variable indicating the HP of engines in use that were first placed in use after 1990 (Adj HP_1990+_). Using these year- and mine-specific variables along with a few other predictors such as season the CO sample was collected, technique used in the CO measurement (detector tube versus bistable), the survey the CO sample came from and use in a mine of long wall mining technique, NIOSH used a regression model to estimate CO concentrations by year and by mine. This regression model was of the form


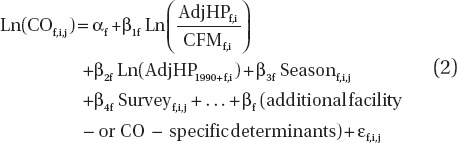


where f indexes mine, i indexes year and j indexes CO sample within a mine and year, Adj HP_f,i_ is the adjusted (for time of use) HP of equipment in Mine f in year i, CFM_f,i_ is the ventilation rate in CFM for Mine f in year i, Adj HP_1990 + f,i_ is 1.0 plus the adjusted HP of diesel equipment purchased in 1991 or later, Season_f,i,j_ is an indicator variable for the season the CO sample was collected, Survey_f,i,j_ is an indicator variable for the survey the CO sample came from, and the ε_f,i,j_ are independent Normal (0,1) random variables. Equation (2) is used only to estimate CO concentrations at the mine face, and it is assumed that yearly CO concentrations varied proportionately throughout a mine. All face area CO measurements were used in fitting this model except for those from the MESA/BoM survey (Table 1). To handle nondetect CO samples, NIOSH/NCI imputed 100 sets of data following Lubin et al. (2004), analyzed all 100 using Equation (2), and then combined these to derive the parameter estimates, standard errors, and 95% confidence intervals using PROC MIANALYZE (SAS). We were able to closely reproduce the NIOSH/NCI parameter estimates and standard errors (Vermeulen et al., 2010b; Table 2).

In applying Equation (2) the MESA/BoM survey data (Table 1) was held out to evaluate the accuracy of their predictions. The remaining CO data was divided into two sets; one set (“DEMS”) comprising DEMS and the feasibility study (Table 1) and the other (“MIDAS”) containing the remaining CO data (mainly MIDAS data). In imputing values to the nondetects they assumed a Log-normal distribution for each of these data sets, stating that “the measurements were approximately lognormally distributed”. However, this appeared to us to not be the case; applying the Shapiro-Wilk test (Royston, 1993) for normality to the log-transformed Midas data led to a firm rejection of the hypothesis that these data were log-normally distributed (*p* = 6 × 10^–23^). The same was true of the DEMS data (*p* = 7 × 10^−8^).

Mine A relied primarily on natural airflow for ventilation, so there are no estimates of airflow rates for this mine. In applying Equation (2) to this mine, Adj HP was substituted for 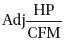. Mine J was also treated as a special case. This potash mine closed in 1993 and consequently it was not included in the 1998–2001 DEMS survey. NIOSH/NCI chose not to develop a CO model for Mine J using Equation (2), but instead applied the determinants for Mine J to the model developed for Mine B, which is another potash mine. In addition, NIOSH/NCI did not use Adj HP_1990+_ in fitting the data for mines A and H, citing collinearity between Adj HP_1990+_ and 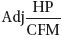


### NIOSH/NCI’s evaluation of the accuracy of their CO model

NIOSH/NCI compared the CO mine-specific model predictions obtained using Equation (2), with CO air concentration measurements from the 1976–1977 MESA/BoM survey that was not used in the modeling, and found that model predictions were generally somewhat lower than the arithmetic means (AM) of the MESA/BoM samples (median relative difference of 33%) (Vermeulen et al., 2010b; Table 3). These results are reproduced in the left part of Table 3. NIOSH/NCI used this finding as a principal support of their claim that the overall evidence suggests that their estimates were likely accurate representations of historical personal exposures. However, in Table 3 we have added the AM for the MIDAS survey samples collected during 1976–1977. The MIDAS samples show much poorer correspondence between the model-predicted results and the AM (median relative difference of −274%) than the MESA/BoM samples. In fact, the model predictions tend to overestimate MIDAS samples over a much wider range of years (data not shown). How can this be, since the model results were based on MIDAS samples and not on MESA/BoM? The answer to this question lies in the fact that the model (Equation (2)) contains a variable to distinguish between the two surveys used in the modeling (DEMS and MIDAS), which allows one to make either “DEMS” estimates or “MIDAS” estimates. The model estimates for 1976–1977 used to compare with the AM from MESA/BoM were “DEMS” estimates, even though the DEMS samples were collected during a much later time (1998–2001). If Vermeulen et al. had used “MIDAS” estimates they would have achieved much better correspondence with the MIDAS AM, but then the agreement with the MESA/BoM data, which had been held out to evaluate the accuracy of their predictions, would have been much poorer. In fact both the DEMS CO samples and the MESA/BoM samples tend to be systematically higher than the MIDAS samples (Vermeulen, 2010b; Table 1), so it would be expected that “DEMS” estimates would predict MESA/BoM data better than “MIDAS” estimates.

**Table 3:** Assessment of differences and relative differences between the mine-specific CO prediction model estimates and the arithmetic means of the CO measurement data for 1976–1977. The estimated CO concentrations and the AM for MESA/BoM come from Table 3 of Vermeulen et al. (2010b). The AM for MIDAS are the average of 100 imputations.

		MESA/BoM (1976–1977)			MIDAS (1976–1977) Estimated CO		
			
Mine	Estimated CO concentration in 1976–1977 (ppm)	*n*	Measured CO concentration AM (ppm)	Relative difference %	*n*	Measured CO concentration AM (ppm)	Relative difference %^a^
B	5.15	90	7.23	29	19	0.76	−579
D	7.98	136	10.50	24	24	4.38	−82
E	10.6	148	8.50	−25	19	2.11	−401
H	3.9	100	7.68	49	7	1.51	−159
I	4.85	122	7.73	37	12	0.99	−389
J	4.36	217	8.09	46	8	4.38	0
Overall median difference			33			−274

AM, arithmetic mean of the CO measurements at the production face collected during 1976–1977; *n*, number of measurements.

^a^ Relative difference is the AM of the measured CO concentrations minus the estimated CO concentration, divided by the AM of the measured concentrations.

It should be noted that this issue does not affect NIOSH/NCI’s yearly estimates of REC (see Equation (3) below) because these estimates depend only upon the ratio of yearly CO estimates to the reference CO level (estimated CO level during 1998–2001). These ratios are the same, regardless of whether “DEMS” estimates or “MIDAS” CO estimates are used.

### The historical relationship between CO and REC

The model for CO specified in Equation (2) was used to predict mine-specific CO values for every year, based on assigned yearly diesel HP values, starting with the first year that a mine used diesel equipment. These yearly CO estimates were converted into mine-specific estimates of personal REC exposures using the formula


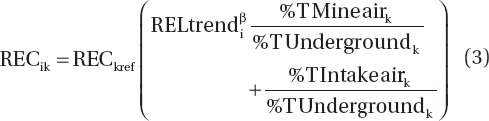


where k indexes job and i indexes year, REC_ik_ = REC personal exposure estimate for year i and job k, REC_kref_ = reference REC exposure estimate assigned to job k, i.e. the 1998–2001 REC estimate, 
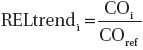
, where CO_i_ is the CO concentration estimated using Equation (2) for year i and CO_ref_ is the CO concentration estimated using Equation (2) for the period 1998–2001, % T Mine air_k_ is the percentage of time a worker in job k is exposed to “mine air”, % T Intake airk is the percentage of time the worker is exposed to “intake air” and % T Underground_k_ is the percentage of time a worker in job k spends underground, and β is the slope parameter in the Ln(REC) - Ln(CO) relationship (Equation (1)). Although not indicated by the notation, all of these quantities are mine specific. While NIOSH/NCI's preferred estimates assumed a seemingly unsupported linear relation between CO and REC (β = 1), they also present REC estimates using their estimated value of β = 0.58 (referred to in Vermeulen et al. (2010b) as CO Model^0.58^).

There are several features of Equation (3) that are worthy of note. It differentiates between areas served by “mine air” and those served by “intake air”. Contrary to what is suggested by the nomenclature, there is no single air that could be identified as “mine air” at any particular time in a mine. For example a job code (k) exposed to 100% “mine air” in 1998–2001 is assumed to be exposed to the reference REC air concentration assigned to that job code (REC_kref_), which would be different for different job codes, rather than uniform.

The concentration of REC in “intake air” does not change with time and is assumed to be the same for all years as measured in the 1998–2001 survey, back to when diesel equipment was first used. Vermeulen et al. (2010b) do not explicitly state how they estimated historical above ground exposures, but apparently they used the same assumption as for “intake air,” i.e. the 1998–2001 estimated level was applied for all years. This impression is reinforced by their statement that their estimation procedure in a few cases for early years of dieselization produced higher REC exposures outside than inside the mine (which caused them to adjust their estimates).

### Our implementation of the NIOSH/NCI CO model

We undertook to construct REC exposures along the same lines as used by NIOSH/NCI but with some important differences. We reimputed values of nondetects for CO using the approach recommended in Lubin et al. (2004). Given the lack of evidence cited earlier that the two CO data sets followed a Log-normal distribution, we used maximum likelihood to fit each of the Log-normal, Gamma, and Weibull distributions to these data sets. (The Midas data were fit on a mine-specific basis, but the NIOSH/NCI data was not subdivided by mine owing to the small number of CO samples.) The Weibull provided the best fit (largest log-likelihood) to the Midas data collected in Mine J, the Log-normal provided the best fit to the Midas data from Mine H, and otherwise (seven cases) the Gamma provided the best fit. We then generated 100 bootstrap samples of censored CO data for each case and, following Lubin et al. (2004), used maximum likelihood to fit the best-fitting model (as noted above) to these bootstrap samples to impute values for the nondetects. A detection limit of 0.3 ppm was used for DEMS samples and a limit of 1 ppm for Midas samples (Vermeulen, 2010b). We then applied Equation (2) to each of 100 sets of imputed CO data, using the same independent variables for each mine as NIOSH/NCI (Vermeulen et al., 2010b; Table 2) for consistency. These results were combined to derive the parameter estimates, standard errors, and 95% confidence intervals using PROC MIANALYZE (SAS). Results from this analysis are shown in Table 4, along with the comparable estimates obtained by NIOSH/NCI (Vermeulen et al., 2010b; Table 2).

Our estimates for the coefficient of Ln (Adj (HP/CFM)) are all positive as are those estimated by NIOSH/NCI (Table 4). Likewise, our coefficients and those of NIOSH/NCI for Ln(Adj HP_1990+_) are all negative, which would be expected if diesel equipment installed after 1990 emitted less CO that older equipment. However, there are differences between our coefficient estimates and those obtained by NIOSH/NCI. Leaving out Mine J, our coefficients for Ln (Adj (HP/CFM)) are insignificant for three mines out of the seven, whereas the NIOSH/NCI coefficients are only insignificant for one mine (Mine G). Our confidence intervals for this parameter are wider than those of NIOSH/NCI which must be due to differences in the method of imputation for the large percentages of nondetected CO samples (30%—61%, Table 4).

**Table 4:** Our results of fitting model for CO (Equation (2)) compared to that of NIOSH/NCI (Vermeulen et al. 2010b, Table 2).

Mine	Our results vs. NIOSH/NCI	*n* (% < LOD)	Intercept	(LN adjusted HP/CFM	LN adjusted HP 1990+	Measurement technique^a^	Long wall mining technique^b^	High period^c^	Season^d^	Survey^e^
A	Our	248 (30%)	−5.16 (−10.61 to 0.28)	2.77 (0.35 to 5.18)	NC	0.09 (−0.39 to 0.56)	NA	NA	0.49 (0.02 to 0.95)	0.15 (−1.10 to 1.39)
	NIOSH/NCI	248 (45%)	NP	1.9 (0.27 to 3.53)	NC	NP	NA	NA	NP	NP
B	Our	447 (38%)	10.18 (4.75 to 15.60)	1.37 (0.46 to 2.29)	−0.06 (−0.21 to 0.08)	−0.6 (−1.01 to −0.18)	NA	NA	0.11 (−0.21 to 0.43)	−1.81 (−2.81 to −0.82)
	NIOSH/NCI	447 (39%)	NP	1.05 (0.52 to 1.58)	−0.04 (−0.13 to 0.04)	NP	NA	NA	NP	NP
D	Our	323 (46%)	8.47 (1.33 to 15.61)	1.22 (−0.16 to 2.61)	−0.17 (−0.36 to 0.02)	NA	NA	NA	−0.07 (−0.68 to 0.54)	−2.35 (−4.04 to 0.66)
	NIOSH/NCI	323 (38%)	NP	0.74 (0.02 to 1.46)	−0.13 (−0.22 to −0.04)	NA	NA	NA	NP	NP
E	Our	207 (34%)	7.53 (−2.29 to 17.34)	1.14 (−0.90 to 3.18)	−0.09 (−0.31 to 0.13)	−0.92 (−1.50 to −0.34)	NA	NA	0.13 (−0.45 to 0.70)	−1.64 (−3.34 to 0.06)
	NIOSH/NCI	207 (20%)	NP	1.29 (0.08 to 2.51)	−0.03 (−0.14 to 0.09)	NP	NA	NA	NP	NP
G	Our	276 (50%)	14.09 (−2.91 to 31.08)	1.98 (−0.63 to 4.59)	−0.32 (−0.68 to 0.04)	NA	NA	NA	0.43 (−0.61 to 1.48)	−2.04 (−4.81 to 0.73)
	NIOSH/NCI	276 (30%)	NP	0.68 (−0.64 to 2.01)	−0.2 (−0.36 to −0.05)	NA	NA	NA	NP	NP
H	Our	2361 (61%)	5.19 (2.72 to 7.65)	0.8 0.46 to 1.13)	NC	NA	−0.58 (−0.83 to −0.32)	1.75 (1.54 to 1.96)	−0.07 (−0.23 to 0.10)	−0.54 (−1.30 to 0.23)
	NIOSH/NCI	2361 (60%)	NP	0.75 (0.45 to 1.05)	NC	NA	−0.55 (−0.77 to −0.32)	1.65 (1.47 to 1.84)	NP	NP
I	Our	2000 (46%)	29.29 (6.44 to 52.14)	3.95 (0.68 to 7.21)	−0.11 (−0.20 to −0.02	−1.62 (−2.18 to −1.05)	1.56 (1.25 to 1.86)	NA	0.15 (−0.13 to 0.43)	−2.25 (−3.53 to −0.98)
	NIOSH/NCI	2000 (46%)	NP	2.72 (1.38 to 4.05)	−0.07 (−0.11 to −0.04)	NP	1.08 (0.95 to 1.02)	NA	NP	NP
J^f^	Our	178 (48%)	1.53 (−14.01 to 17.07)	0.32 (−2.65 to 3.29)	NA	−0.9 (−1.62 to −0.17)	NA	NA	0.22 (−0.38 to 0.82)	NA

NA, Not applicable – variable was not included in the model for that facility; NC, Not calculated due to collinearity; NP, Not provided. Vermeulen et al. (2010b) indicated that these parameters were part of the model but parameter estimates were not reported in the article.

^a^ Measurement technique was codes a 1 for detector tube, 0 for bistable.

^b^ Long wall mining was codes as 1 for yes and 0 for no.

^c^ High Period was codes as 1 for yes and 0 for no.

^d^ Season was coded as 0 for season 1 and 1 for season 2.

^e^ Survey was coded as 1 for Midas and 0 for NIOSH or ANIOSH.

^f^ Vermeulen et al. (2010b) used the model fit to the Facility B data for predictions of CO for Facility J.

Recall that there were no DEMS samples available for Mine J, and consequently NIOSH/NCI applied parameter values for Mine B to determinants from Mine J to estimate CO exposures in Mine J. Our parameter values for Mine J in Table 4 are based only on the MIDAS samples available for this mine.

NIOSH/NCI’s decision not to include AdjHP_1990+_ in their model for Mines A and H due to collinearity made a large difference in their estimates of the parameter β_1f_ for 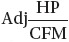 We ran our model for these mines both excluding and including Adj HP_1990+_. Including Adj HP_1990+_ in the model for Mine A caused β_1f_ to become nonsignificant, and in the case of Mine H caused β_1f_ to become negative, while β_2f_ remained significantly negative. In our alternative estimates of REC (shown below), for consistency we follow the NIOSH/NCI approach in omitting Adj HP_1990+_ from the model for Mines A and H. Nevertheless, we believe the results for these mines are more uncertain because of this feature.

### Our implementation of the NIOSH/NCI method for estimating historical REC exposures

To estimate REC, it was not possible for us to apply Equation (3) because we did not know precisely which mine areas were assumed by NIOSH/NCI to receive “mine air” versus “surface air.” However, rather than distinguishing “mine air” from “surface air” and assuming “surface air” does not change with time over the period of operation of a mine, it seems to us that it would be at least as reasonable to assume surface dieselization followed about the same trend as underground dieselization. This assumption leads to a simpler form of Equation (3),


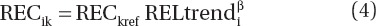


in which the exposure in job k for year i is simply the exposure for the reference year (1998–2001) multiplied by the trend estimated from the CO data, raised to the power, β, that reflects the relationship between CO and REC. Instead of assuming a linear relationship between CO and REC (β = 1) as NIOSH/NCI did in their preferred exposure assessment, we used the relationship we obtained from the DEMS data.

Ratherthanusingonlybestfitparameterstoobtainasingle group of estimates of yearly REC exposures, as was done by NIOSH/NCI, we used the variance-covariance matrix from our analysis to develop bounds for REC estimates. We assumed that the coefficients for 
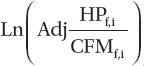
 and Ln(Adj HP_1990 + f,i_) (i.e. β_1f_ and β_2f_), obtained from application of Equation (2), have a bivariate normal distribution, simulated values for β_1f_ and β_2f_ by simulating from the bivariate normal distribution having the point estimates of β and β as the mean values and variance-covariance matrix given by the estimated variance-covariance matrix from the PROC MIANALYZE (SAS) procedure. We also similarly generated a normal distribution for β, the coefficient of the relationship between Ln(REC) and Ln(CO), based on the results of our analysis of the DEMS data reported earlier (β = 0.30, 95% CI: (0.061, 0.54)). We sampled from the joint distribution of β_1f_ and β_2f_ 100 times and, applying Equation (2), generated 100 sets of yearly CO values for each mine. To each of these 100 sets we applied Equation (4) to estimate yearly REC values, using a simulated value of β. It should be noted that the other coefficients in Equation (2), other than β_1f_ and β_2f_, relate to year-independent explanatory variables, and consequently their values cancel out when applying Equation (4).

**Figure 4:**
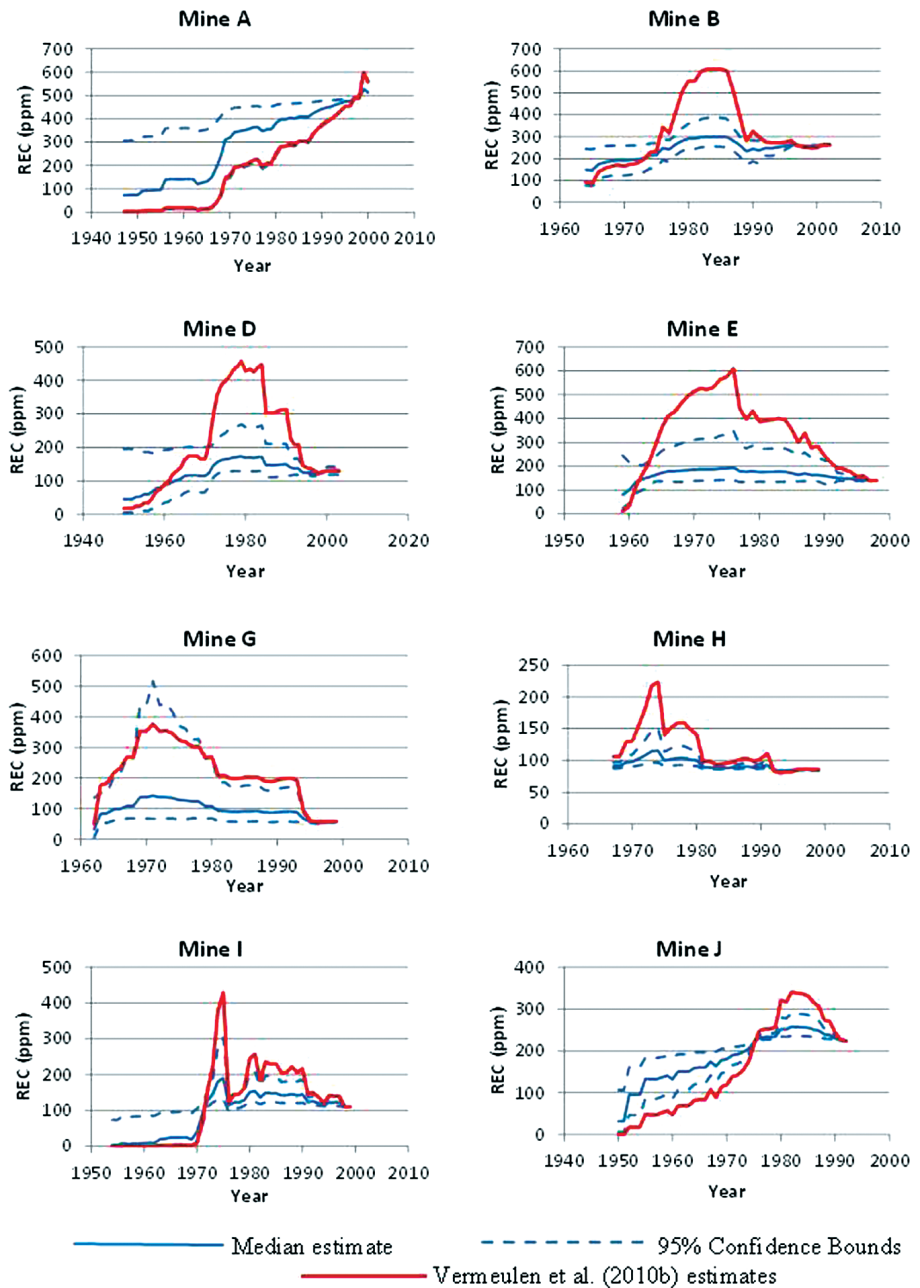
A comparison of our median estimates and 5th and 95th percentiles for production workers exposure to REC with NIOSH/NCI's preferred estimates (Vermeulen et al. 2010b, Figure 3). For consistency the same parameters were used in our implementation of the CO model as were used by NIOSH/NCI. Likewise, following NIOSH/NCI, our model for Mine J is based on parameter values from Mine B.

Figure 4 shows the median values of REC historical predictions (µg/m^3^) for the mine operator, along with the corresponding 5th and 95th percentiles. Mine A had no mine operator and therefore, following NIOSH/NCI, results for the loader operator are depicted. Also shown are our recreation of NIOSH/NCI’s preferred estimates (Vermeulen et al., 2010b; Figure 4), which are based on assuming a linear relationship between CO and REC (β = 1). For consistency, we included the same parameters in the model for each mine as NIOSH/NCI (Table 4), and for Mine J, we used the model for Mine B, just as was done by NIOSH/NCI.

The differences between our 5th percentiles and our 95th percentiles in Figure 4 are quite large for some years, particularly in the earlier years (e.g., Mines A, D, and I), indicating a greater uncertainty regarding REC exposures in these years. The NIOSH/NCI estimates tend to be larger than our median estimates in some mines (e.g. B, D, and E) and smaller in others (e.g. A). Although our curves are based on Equation (4) and NIOSH/NCI’s are based on Equation (3), this should account for very little of the differences since mine operators were assumed to spend at least 85% of their time at the mine face, which was assumed by NIOSH/NCI to be served by “mine air.” This is further supported by the fact that our curves agree closely with NIOSH/NCI’s for the years 1998–2001. We note that all of the curves for a given mine have the same general shape. This is because all are based on the same estimates of HP, as we had no data upon which to estimate the uncertainty in these estimates. Consequently, the ranges shown in Figure 4 do not represent the total uncertainty in REC estimates.

## Discussion

Estimating historical exposures to a cohort for use in an epidemiological study is generally a very difficult and uncertain undertaking. This is particularly true in this case, as NIOSH/NCI only had REC samples (their sur rogate for DE) available primarily for the period 1998 and to 2001, whereas diesel equipment was first used in the mines beginning in the 1940s through 1960s. To project REC exposures back 50+ years they opted to rely upon CO as a surrogate for REC. Although samples of CO were fairly numerous during 1976–2001, there were a very limited number available prior to 1976 (Table 1) and these early exposures may be particularly important in evaluating lung cancer which typically has a long latency period. A large percentage (45%) of the CO samples were below the detection limit, and NIOSH/NCI used imputed values for these samples in their analysis. To account for the lack of CO samples during earlier times, NIOSH/NCI used a second surrogate, HP (adjusted for usage), and divided by CFM (mine exhaust rate) to estimate levels of the surrogate CO. Although this “double surrogate” approach was largely dictated by the data available, still the significant uncertainties in the resulting REC estimates need to be recognized.

NIOSH/NCI assumed a linear relationship between CO and REC (REC ∼ CO^1.0^ ) in their preferred set of REC exposure estimates, despite estimating a relationship of (REC ∼ CO^0.58^) from their data. The only support they provided for this decision was a reference to Yanowitz et al. (2000). However, when we analyzed data from this article we obtained an exponent less than 1.0, whose 95% confidence interval did not include 1.0 (Figure 1). Moreover, we could not verify the exponent of 0.58 claimed by NCI/ NIOSH. Instead we obtained exponents in the range of 0.39–0.44 (Table 2) when we attempted to replicate their analysis and a value of 0.30 when we applied alternative (and, we believe, better) methods for imputing values for the nondetected CO samples. Thus, the linear relationship that NIOSH/NCI assumed between historical CO levels and REC levels does not appear to be supported by the literature or their data.

The effect of using an exponent less than 1.0 to estimate the CO-REC relationship (Equations (3) or (4)) compared to using an exponent of 1.0 depends upon whether the estimated yearly CO concentration is less than or greater than the reference concentration (CO_ref_, estimated for the period 1998–2001). For those yearly CO concentrations less than the reference value, using an exponent less than 1.0 will give a larger estimate of REC than using 1.0 as the exponent, and *vice versa*. If, as is approximately the case in several of the mines, the estimated CO starts at a low value with the beginning of dieselization, increases to a maximum as diesel equipment is added and then begins to decrease (due to a plateau or decrease in HP coupled with improved ventilation – see Figure 1 of Vermeulen et al., 2010b and Figure 3 of this article) down to the reference value in 1998–2001, use of an exponent less than 1.0 will cause the yearly REC estimates to be larger up to the year in which the CO estimate first exceeds the reference value, and to be smaller in subsequent years, compared to using an exponent of 1.0. The effect of such a change upon the outcome of the epidemiological analyses (Attfield et al., 2012; Silverman et al., 2012) is difficult to predict.

Clark et al. (1999) examined CO and PM data collected from seven different test sites, representing 11 different engine types, many operated under different loading conditions, and concluded that “… there is no universal relationship between CO and PM. [The] … data …. suggests that the CO/PM relationship is unique for each engine type and perhaps for each engine.” Diesel oxidation catalysts (DOCs) began to be installed on diesel equipment used in mines in the 1970s and 1980s. DOC is an after-treatment technology for diesel-powered equipment that can efficiently convert CO in the exhaust stream to CO_2_, and thereby greatly decrease ratios of CO/REC (Hesterberg et al., 2012). NIOSH/NCI apparently did not use any information on the extent of DOCs usage, which likely made CO a less reliable indicator of REC in their analyses.

The relationship between HP and CO assumed by NIOSH/NCI is similarly subject to doubt. The article by Yanowitz et al. (2000) which NIOSH/NCI used to support their relation between REC and CO, also has several hundred simultaneous measurements of HP and CO. Using these data, the slope of the regression line of Ln(HP) on Ln(CO) is only borderline significant (*p* = .05) and the data indicate a large variation around the fitted line (*r*^2^ = 0.01, Figure 2).

NIOSH/NCI applied inventories of diesel-powered equipment used underground, along with historical data on mine ventilation rates, to develop yearly estimates of HP of equipment used in the mines and air flow rates in the mines. The underlying information on these estimates was not available to us, so it was not possible for us to evaluate their uncertainty. But we expect that it is considerable. For example NIOSH/NCI state that their estimates were based on inventories of diesel equipment that go back as far as the early 1970s, although these inventories were rarely available for the 1980s (Vermeulen et al., 2010b). However, their HP estimates go back to when diesel equipment was first introduced into these mines (1947–1967), which suggests that these earlier estimates may be particularly uncertain.

NIOSH/NCI assumed that estimates of above ground REC exposures were time-independent and assigned the exposures measured during 1998–2001 to all past years in which diesel equipment was used in a mine. This assumption seems highly implausible and consequently estimates of above ground exposures appear to be particularly uncertain. It seems to us that it would be more plausible to assume that surface dieselization and the resulting exposures followed the same trend as underground dieselization and exposures.

We implemented alternatives to the NIOSH/NCI preferred estimates of REC using their general approach but making several of what we believe to be improvements to their approach. Rather than using just best estimates of parameters, our approach took into account the statistical uncertainty in these parameter estimates to derive ranges for REC exposures. Other improvements included: (i) an improved method for imputing values to nondetected CO samples; and (ii) a data-driven approach to the REC vs. CO relationship rather than assuming a linear relationship. Our REC estimates for mine operators are shown in Figure 4 where they are also compared to NIOSH/NCI’s preferred estimates. These figures show substantial differences between our estimates and those of NIOSH/NCI. For most mines the preferred NIOSH/NCI estimates do not lie completely within the confidence bands established in our analysis.

In addition to their preferred REC estimates, which are based on an assumed linear relationship between CO and REC (β = 1), NIOSH/NCI also developed two sets of alternative estimates. One alternative estimate was based on 5-year average measured CO levels from the MIDAS survey, which only covered the period post-1975, and earlier CO exposures were estimated using mine-specific changes in Adj HP/CFM relative to the 1976 values. The assumption of a linear relationship between CO and REC (β = 1) was retained. The other alternative estimate was based on the estimate of β they obtained from their analysis (β = 0.58). However, we were unable to duplicate this value, obtaining instead smaller values (Table 2), and, in particular, after implementing our preferred method for assigning values to nondetected CO measurements, obtained a best estimate of β = 0.30. In their preferred estimates, as well as in these alternatives, NIOSH/NCI used only point estimates of parameters and did not take into account their statistical variability.

To access the reliability of their CO model, NIOSH/NCI compared predictions from their CO model to CO data from the 1976–1977 MESA/BoM survey, which was not used in the modeling. The found a median relative difference of 33%, which NIOSH/NCI interpreted as supporting the accuracy of their model. However, their CO model contained a terms for survey, which allows one to make estimates specific to a survey (DEMS or MIDAS). The comparison reported by NIOSH/NCI was based on the DEMS survey, even though CO measurements from this survey were all made 20+ years after those in the MESA/BoM survey. A comparison of the model predictions used by NIOSH/NCI (based on the DEMS survey) with MIDAS data collected during 1976–1977 reveals a much poorer fit (mean relative difference of –274%) even though the MIDAS data were used in developing the CO model.

The ranges for our REC estimates shown in Figure 4 should not be interpreted as showing the full uncertainty of REC exposures. For example, they do not include any information on the uncertainty of the HP and CFM estimates, which we expect is considerable. Rather they are presented to show how moderate changes to NIOSH/NCI’s approach (which, we think, are reasonable and provide improvements to the NIOSH/NCI approach) can affect their estimates.

NIOSH/NCI’s estimates of REC exposures in Mine J are particularly uncertain as there were no REC samples available for this mine, which closed in 1993 before the DEMS survey. NIOSH/NCI estimated REC exposures for this mine by applying parameter values in their CO model (Equation (2)) obtained from Mine B to determinants from Mine J (both were potash mines), and estimating 1993 REC exposures in Mine J using REC data obtained from mine B.

Other uncertainties come into play when one attempts to apply these exposure estimates to individuals who worked in the mines. For example, did the worker use a respirator and if so, how often, and how effective was the respirator? In a tabulation that accounted for 84% of the 21,805 MIDAS samples collected by MSHA from April 1, 1988 through 1992, the percentage of workers at various underground jobs who were using respirator equipment ranged between 38% and 82% (Watts & Parker, 1995; Table 4). This suggests that respirator use was common among underground miners during this period.

Some notion of the uncertainty in the NIOSH/NCI estimates can be obtained from comparing estimates we made using what we believe to be improvements to their methodology to their preferred estimates (Figure 4). But we need to emphasize that the range in our estimates illustrated by the confidence bounds do not incorporate all of the uncertainties and perhaps not even the most important ones (e.g. the assignment of HP by years and the assumption of a reliable relationship between HP and CO). Perhaps the general form of NIOSH/NCI’s estimation procedure (e.g. using CO as a surrogate for REC and HP as a surrogate for CO) was about the best that could be devised given the limitations in the data available. However, this should not prevent one from acknowledging the significant uncertainties in the derived exposure estimates, and attempting to account for them in any endeavor to use the estimates to evaluate patterns of disease among the miners who worked in these mines.

## Footnotes

^1^ Stewart et al. (2010) state that the override were “assigned the AM of the measurements of all the jobs in the fresh air in the corresponding underground facility, although they were based on fewer than five measurements,” although they do not state how samples were determined to be of fresh air, and some of the assigned override exposures were far higher than any measured above ground.

^2^ Imputed using a maximum likelihood approach, assuming the reported median detection limit of 0.3 ppm for all CO samples (Vermeulen 2010a) and assuming, as did Vermeulen et al. (2010a), that mine-specific CO values are log-normally distributed.
